# First-time rest-stress dynamic whole-body ^82^Rb-PET imaging using a long axial field-of-view PET/CT scanner

**DOI:** 10.1007/s00259-023-06242-z

**Published:** 2023-04-27

**Authors:** Federico Caobelli, Sigrid Seibel, Korbinian Krieger, Carola Bregenzer, Marco Viscione, Angela Filipa Silva Mendes, Hasan Sari, Lorenzo Mercolli, Ali Afshar-Oromieh, Axel Rominger

**Affiliations:** 1 Department of Nuclear Medicine, Inselspital, Bern University Hospital, University of Bern, Freiburgstrasse 18, 3010 Bern, Switzerland; 2 Advanced Clinical Imaging Technology, Siemens Healthcare AG, Lausanne, Switzerland

Recently, long axial field-of-view (LAFOV) PET/CT scanners have been introduced, which yield a substantial increase in sensitivity compared to standard scanners [1, 2] and allow for acquiring whole-body dynamic imaging [3]. This may constitute the basis for a new diagnostic concept in cardiovascular imaging, e.g. in patients with suspected or known coronary artery disease (CAD).

Recent evidences highlighted the interconnection between ischemic heart and other organs such as brain [4] or kidney [5, 6], consistent with the concept that CAD is linked to a systemic disease.

We performed a whole-body rest/stress perfusion with [^82^Rb]Cl PET using a Biograph Vision Quadra™ PET/CT (Siemens Healthineers, Knoxville, TN, USA). A 57-year-old female patient presented with atypical chest pain, without history of CAD nor known cardiovascular risk factors. Both at rest and during stress, images were acquired over 7 min in list mode immediately following an intravenous administration of 407 MBq (11 mCi) [^82^Rb]Cl. Pharmacological stress was induced with Regadenoson (400 mcg).

Images were reconstructed using a dedicated image-reconstruction prototype (e7-tools, Siemens Healthineers) in a dynamic fashion in cine-mode and further displayed as static images corresponding to different time intervals from the injection. In the upper row of the image, rest imaging is displayed featuring 0–30 s (a), 30–60 s (b), 60–120 s (c) and 120 s–7 min (d) from the injection, respectively. In the bottom row, for stress imaging: 0–30 s (f), 30–60 s (g), 60–120 s (h) and 120 s–7 min (i), respectively. Homogeneous perfusion of the myocardium was shown (e, rest and l, stress).

The activity flows rapidly through the peripheral venous system, also distributing in the pulmonary vessels (a,f). Right after, the arterial vessels of the whole body and both kidneys can be visualized (b,g). Then, salivary glands, thyroid, spleen and liver can be seen. Also, starting from 60 s post-injection the LV-myocardium is fully visualized (c,h). Time-activity curves on LV-Myocardium and on both kidneys could be derived from dynamic data (m) and showed an increase in myocardial blood flow after injection of Regadenoson. Despite a similar appearance on static images, renal flow was significantly lower for the right kidney compared to the left organ. Furthermore, while the flow was similar on rest and during Regadenoson stress on the left side, there was a further slight decrease in flow after injection of Regadenoson on the right side.

The images reconstructed at the latest time point show increased spleen-to-liver activity ratio in rest conditions than in stress (d, l, red arrows). The splenic switch-off is known when using Regadenoson as stressor [7] and with whole-body imaging it can be used to identify inadequate vasodilatory response. Renal function may also be effectively evaluated, consistent with recent reports [8].

^82^Rb-whole body PET imaging on LAFOV scanners also bears the potential for insightful investigations on the relationship between perfusion status and function in other organs, e.g. hyperperfusion in the thyroid that can be associated to a thyroiditis [9]. Unfortunately, ^82^Rb PET did not show as suitable to assess brain perfusion [10, 11].

The use of LAFOV PET/CT scanners in whole-body perfusion imaging goes beyond the concept of “pretty images”. Besides an improvement of image quality and a substantial reduction of administered dose, the simultaneous dynamic whole-body acquisition opens the door for the investigation of the intimate connections of various organs in the assessment of patients with suspected CAD.
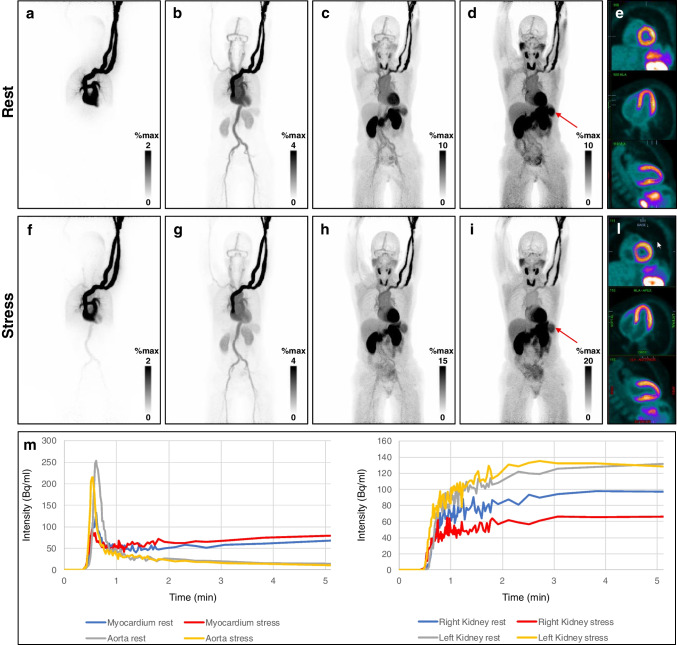


## Data Availability

Data will be made available upon reasonable request.

## References

[1] Alberts I, Hünermund JN, Prenosil G, Mingels C, Bohn KP, Viscione M, Sari H, Vollnberg B, Shi K, Afshar-Oromieh A, Rominger A (2021). Clinical performance of long axial field of view PET/CT: a head-to-head intra-individual comparison of the Biograph Vision Quadra with the Biograph Vision PET/CT. Eur J Nucl Med Mol Imaging.

[2] Prenosil G, Sari H, Fürstner M, Afshar-Oromieh A, Shi K, Rominger A, Hentschel M (2022). Performance characteristics of the Biograph Vision Quadra PET/CT system with a long axial field of view using the NEMA NU 2–2018 standard. J Nucl Med.

[3] Sari H, Mingels C, Alberts I, Hu J, Buesser D, Shah V, Schepers R, Caluori P, Panin V, Conti M, Afshar-Oromieh A, Shi K, Eriksson L, Rominger A, Cumming P (2022). First results on kinetic modelling and parametric imaging of dynamic ^18^F-FDG datasets from a long axial FOV PET scanner in oncological patients. Eur J Nucl Med Mol Imaging.

[4] Thackeray JT, Hupe HC, Wang Y, Bankstahl JP, Berding G, Ross TL, Bauersachs J, Wollert KC, Bengel FM (2018). Myocardial inflammation predicts remodeling and neuroinflammation after myocardial infarction. J Am Coll Cardiol.

[5] Werner RA, Hess A, Koenig T, Diekmann J, Derlin T, Melk A, Thackeray JT, Bauersachs J, Bengel FM (2021). Molecular imaging of inflammation crosstalk along the cardio-renal axis following acute myocardial infarction. Theranostics.

[6] Fukushima K, Javadi MS, Higuchi T, Bravo PE, Chien D, Lautamäki R, Merrill J, Nekolla SG, Bengel FM (2012). Impaired global myocardial flow dynamics despite normal left ventricular function and regional perfusion in chronic kidney disease: a quantitative analysis of clinical 82Rb PET/CT studies. J Nucl Med.

[7] Saad JM, Ahmed AI, Han Y, El Nihum LI, Alahdab F, Nabi F, Al-Mallah MH. Splenic switch-off in regadenoson ^82^Rb-PET myocardial perfusion imaging: assessment of clinical utility. J Nucl Cardiol. 2023. 10.1007/s12350-022-03158-3. Epub ahead of print. 10.1007/s12350-022-03158-3 36607537

[8] Langaa SS, Mose FH, Fynbo CA, Theil J, Bech JN (2022). Reliability of rubidium-82 PET/CT for renal perfusion determination in healthy subjects. BMC Nephrol.

[9] Hiromatsu Y, Ishibashi M, Nishida H, Kawamura S, Kaku H, Baba K, Kaida H, Miyake I (2003). Technetium-99 m sestamibi imaging in patients with subacute thyroiditis. Endocr J.

[10] Zünkeler B, Carson RE, Olson J, Blasberg RG, Girton M, Bacher J, Herscovitch P, Oldfield EH (1996). Hyperosmolar blood-brain barrier disruption in baboons: an in vivo study using positron emission tomography and rubidium-82. J Neurosurg.

[11] Brooks DJ, Beaney RP, Lammertsma AA (1984). Quantitative measurement of blood-brain barrier permeability using rubidium-82 and positron emission tomography. J Cereb Blood Flow Metab.

